# A Human Long Non-coding RNA LncATV Promotes Virus Replication Through Restricting RIG-I–Mediated Innate Immunity

**DOI:** 10.3389/fimmu.2019.01711

**Published:** 2019-07-19

**Authors:** Jingjing Fan, Min Cheng, Xiaojing Chi, Xiuying Liu, Wei Yang

**Affiliations:** NHC Key Laboratory of Systems Biology of Pathogens, Institute of Pathogen Biology, Chinese Academy of Medical Sciences and Peking Union Medical College, Beijing, China

**Keywords:** interferon-regulated lncRNAs, retinoic acid-inducible gene 1, innate immune response, hepatitis C virus, Zika virus

## Abstract

Pattern recognition receptors sense pathogen components and initiate the host antiviral innate immune response, such as inducing interferons (IFNs). Long non-coding RNAs (lncRNAs) are emerging regulators of multiple biological processes. However, their role in antiviral response, especially through regulating the human innate immune, is largely unexplored. Here we characterized that lncATV, a human specific lncRNA, was up-regulated upon type I/III IFN stimulations and virus infection. LncATV was cytoplasmic localized and relatively high expressed in human monocytes, erythroleukemia cells and hepatoma cells. Notably, lncATV knockdown significantly inhibited the replication of multiple RNA viruses, such as hepatitis C virus, Zika virus, Newcastle disease virus, and Sendai virus. Mechanistically, RIG-I antiviral signaling and IFN effective pathway were enhanced when lncATV expression was knocked down but inhibited by overexpressed lncATV. RNA immunoprecipitation results demonstrated an association between LncATV and RIG-I. Collectively, our findings reveal the functional role of a novel human specific lncATV as a regulatory lncRNA restricting virus associated innate immune response.

## Introduction

Host response to viral infection consists of innate and adaptive defenses. The innate immune response is considered the first line of antiviral defense because it is active even at the same time of infection initiation (1, 2). Recent findings also indicate that innate immunity activation plays pivotal roles in the regression of tumors (3). Pattern recognition receptors (PRR) constitute a large part of the host antiviral innate immune system (4). Several families of PRRs, such as retinoic acid inducible gene I–like receptors (RIG-I–like receptors, RLRs), are known to play a crucial role in host defense against RNA viruses (5). As a member of RLRs, RIG-I primarily detects viral RNA molecular signatures. RIG-I binding of virus-derived RNA results in ATP binding, release of the auto-inhibited tandem caspase activation recruitment domains (CARDs), formation of a tetrameric complex of RIG-I, interaction with the CARD domain of the mitochondria activating signaling protein (MAVS), and subsequent activation of downstream signaling to induce the production of type I or type III interferons (IFNs) and IFN-independent antiviral responses (6–8). However, precise regulation of the innate immune pathway is critical to both robust immunity to pathogens and limiting excessive autoimmunity to host itself.

So far, most studies on the host innate response to virus infection typically focus on protein-coding genes. However, non-coding RNAs (ncRNAs) are substantially transcribed from human genome, and the roles of many of these ncRNAs remain enigmas (9, 10). Long non-coding RNAs (lncRNAs), which are defined as being longer than 200 nt in length, often show spatiotemporally restricted expression patterns and functionally regulate a variety of biological processes in cells and organ systems (11–13). LncRNAs as novel players in regulation of antiviral immune response were recently reported. Usually, RIG-I is able to discriminate pathogenic RNA from self-RNA, including host-derived lncRNAs. The underlying mechanism of RIG-I's specificity and activation must be meticulously tuned through types of negative or positive regulations. Specific host lncRNAs can be induced or reduced to modulate innate and adaptive immune responses through various target interactions, such as lncRNA-protein interaction and hence affect the immunogenic regulation at various stages of gene expression. Jiang and colleagues have shown that lnc-Lsm3b, a mouse specific type I IFN-induced lncRNA, has the ability to bind to RIG-I and prevents further activation of RIG-I by acting as a decoy for RIG-I (14). LncRNA-Cox2 is induced in murine bone marrow derived macrophages by lipopolysaccharide, and modulates a panel of immune response genes, including chemokines and IFN-response genes (15). NRAV is a lncRNA promoting influenza A virus replication by negatively regulating the initial transcription of multiple interferon-stimulated genes (ISGs) (16). In addition, lnc-DC promotes differentiation of monocytes to conventional dendritic cells by binding to and dephosphorylation the STAT3 transcription factor (17). These evidences suggest that lncRNAs indeed participate in the regulation of infection and innate immune response, though the number reported so far is extremely limited compared with the protein-coding genes. Therefore, extensive identification of the novel functional lncRNAs is urgently needed.

To address this issue, we systematically characterized the IFN-regulated lncRNAs in type I or type III IFN stimulated human hepatoma cells. By screening antivirus activity, we focused on the functional characterization of a novel human specific IFN-induced lncRNA, lncATV, for its regulation of multiple RNA virus infections. Furthermore, we provide mechanistic data showing that lncATV participates in the RIG-I-mediated antiviral innate immune response.

## Materials and Methods

The detailed information of cells, reagents, virus infection, plasmids, RNA interference, RNA pull-down assay, In-Cell Western, luciferase reporter assay, quantitative real-time reverse transcription-PCR, Western blotting, confocal microscopy, fluorescence *in situ* hybridization, and statistical analysis is described in the Supplementary Information.

## Results

### LncATV Is Induced by IFNs and Virus Infections

**Figure 1:**
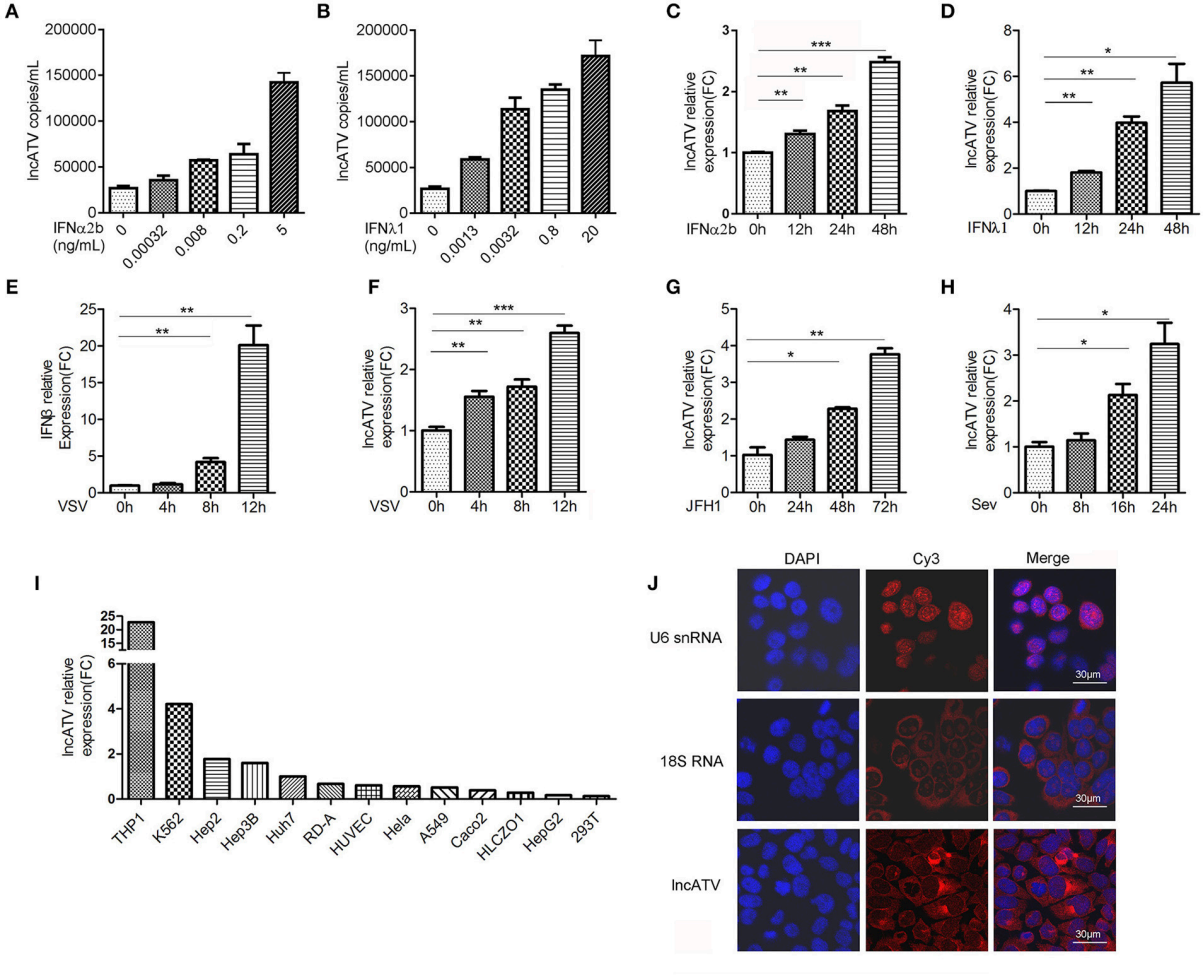
LncATV is induced by IFNs and virus infections. **(A,B)** Endogenous LncATV expression levels were analyzed by absolute qRT-PCR in IFNα2b **(A)** or IFNλ1 **(B)** stimulated Huh7 cells at different IFN dosages. The treatment duration was set to 48 h. **(C,D)** Endogenous LncATV expression levels were analyzed by relative qRT-PCR in IFNα2b **(C)** or IFNλ1 **(D)** stimulated Huh7 cells at different treatment timepoints. The treatment concentration is 1 ng/mL for IFNα2b and 25 ng/mL for IFNλ1. **(E)** The levels of IFNβ mRNA were determined by qRT-PCR in Huh7 cells after VSV infection. **(F–H)** The expression levels of LncATV were analyzed by qRT-PCR in VSV **(F)**, JFH-1 HCVcc **(G)**, or SeV **(H)** infected Huh7 cells at various infection time points. **(I)** Quantitative RT-PCR determination of the endogenous lncATV abundance in multiple tissue type derived cell lines. The relative level of lncATV in Huh7 cells was set as 1. **(J)** RNA FISH detecting the subcellular localization of endogenous lncATV molecules in Huh7 cells. U6 snRNA or 18S RNA specific probes were used as control to indicate nucleus or cytoplasm localization, respectively. Red signals show Cy3-labeled RNAs, and DNA (blue) was stained with DAPI. A representative image is shown. Scale bars, 30 μm. Data are representative of three independent experiments and plotted as the mean ± s.d. ^*^P < 0.05, ^**^P < 0.01, ^***^P < 0.001 vs. the control group.

Type I and type III IFNs are induced at the acute initial infection and play important roles in restricting viral production in infected cells by inducing ISGs (18–20). Hundreds of ISGs have been identified, though IFN-stimulated lncRNAs are largely unclear. To systematically investigate the regulation of lncRNAs by IFNs, we first chose IFNα2b and IFNλ1 to optimize their treatment concentrations and time durations in Huh7 human hepatoma cells, due to their possible different potency. Using luciferase reporter hepatitis C virus (Jc1-HCVcc) infection model and the induction of MxA and IFIT1 as readouts, we determined the treatment conditions for subsequent functional experiments as follows: 1 ng/mL for IFNα2b and 25 ng/mL for IFNλ1 and both for 8–24 h (Supplementary Figures 1A–F). We then systematically elucidated the changes in host lncRNA expression in Huh7 cells stimulated by type I or type III IFNs using microarrays. Of those lncRNAs found to be differentially expressed (fold change >2.0) in response to IFNα2b or IFNλ1, 272 upregulated, and 631 downregulated lncRNAs were similarly affected by both types of IFNs, respectively (Supplementary Figure 2A). The circos plots indicated differentially expressed lncRNAs and mRNAs on each chromosome (outer track: lncRNAs; inner track: mRNA genes) (Supplementary Figure 2B). Distinct from significant induction of mRNA, regulations of lncRNAs by IFNs were much milder. Several top-ranked differentially expressed lncRNAs were chosen for knockdown with small interfering RNAs (siRNAs) to evaluate their potentials in the regulation of HCV infection in Huh7 cells. Interestingly, a lncRNA (boxed with blue dashed line) showed significant proviral function because HCV infection level was decreased to 20–30% after siRNA knockdown (Supplementary Figure 2C). Therefore, we focused on this lncRNA (ENST00000458044.1) for further characterization and it was termed as lncATV in this study. LncATV is located in chromosome 1 with a single exon (Supplementary Figure 2D). LncATV is a human species specific lncRNA (Supplementary Table 2) and its biological function was never previously reported. We first validated the regulation of lncATV expression by IFNs in Huh7 cells using qRT-PCR (Figures 1A–D). IFNα2b and IFNλ1 significantly up-regulated the expression of endogenous lncATV in a temporal and dose-dependent manner. Furthermore, we investigated whether virus infection could regulate lncATV expression. Vesicular stomatitis virus (VSV) infection triggered innate immune activation by inducing IFNβ expression (Figure 1E). Meanwhile, we found that infection with three RNA viruses, VSV, HCV, and Sendai virus (SeV), all up-regulated lncATV in Huh7 cells (Figures 1F–H). LncRNAs normally have distinct expression pattern in different species and tissue types. We noted that endogenous lncATV level variated in cell lines from different tissue sources, and highly expressed in human monocytes, erythroleukemia cells, and some hepatoma cells (Figure 1I). To gain more insight of lncATV's function, we examined its localization by performing RNA fluorescent *in situ* hybridization (FISH) in Huh7 cells. This analysis revealed that lncATV was predominantly enriched in the cytosol (Figure 1J). As expected, the control 18S RNA was localized to the cytosol, while U6 snRNA was confined to the nucleus (Figure 1J).

### LncATV Is Essential for Effective Replication of RNA Viruses in Human Cells

**Figure 2:**
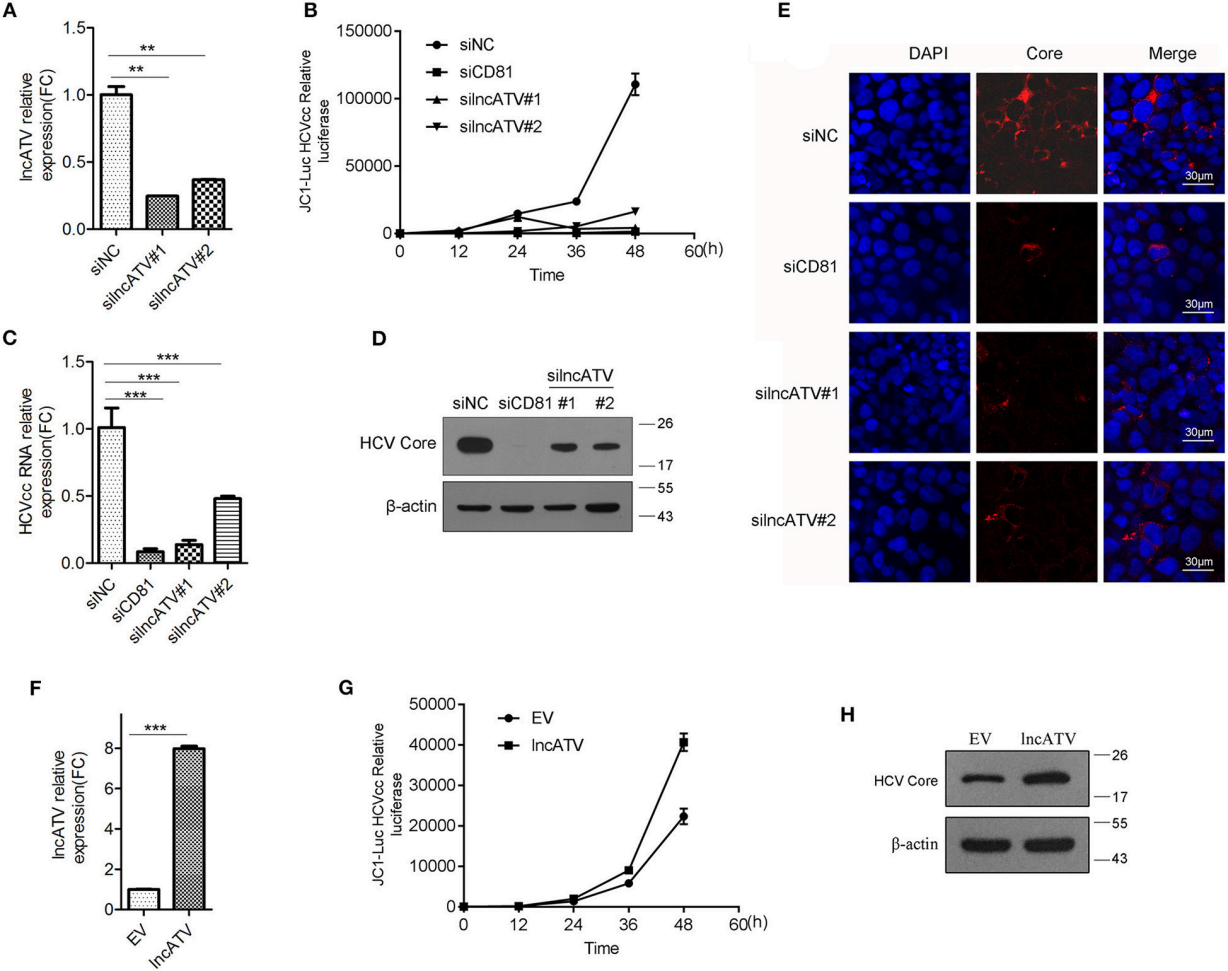
LncATV promotes HCV infection. **(A)** Huh7 cells were transfected with negative control siRNA (siNC) or siRNAs targeting lncATV. Gene silencing effects were measured by qRT-PCR. **(B)** Huh7 cells were transfected with various siRNAs at 24 h prior to Jc1-Luciferase reporter HCVcc infection. Luciferase activity was assayed at different timepoints post-infection. **(C–E)** As described in **(B)** except for the reporter HCVcc replaced by JFH-1 HCVcc. Intracellular HCV RNA **(C)** and viral core protein **(D)** were quantified by qRT-PCR with specific primers and Western blotting with indicated antibodies. Intracellular HCV core expression was detected by immunofluorescent staining **(E)**. Scale bars, 30 μm. **(F–H)** Overexpression of lncATV exerts anti-HCV effect. Huh7 cells were transfected with lncATV expression plasmid or empty vector (EV) as a negative control at 24 h prior to Jc1-Luciferase reporter HCVcc infection. At 72 h or indicated timepoints post-infection, levels of lncATV **(F)**, luciferase activity **(G)**, and HCV core protein **(H)** were measured by qRT-PCR and Western blotting. The data represented mean ± SD (*n* ≥ 3). ^**^P < 0.01, ^***^P < 0.001.

To further determine the functionality of lncATV in virus infection, we knocked down the endogenous lncATV using small interference RNAs (siRNAs) in Huh7 cells and the specific repression of lncATV expression by siRNAs was confirmed (Figure 2A). Strikingly, lncATV silencing led to a significant inhibition of HCV infection with the reduction of Jc1-luciferase reporter HCVcc (Figure 2B) and JFH-1 HCVcc at both the RNA (Figure 2C) and protein (Figure 2D) levels, or the positively stained HCV core antigen (Figure 2E). Here, a siRNA targeting the HCV entry receptor CD81 was used as a positive control to eliminate HCV infection (Figures 2B–E). In stark contrast, the increased HCV propagation from lncATV-overexpressing cells was further observed by reporter assay and Western blotting using HCV Core antibody (Figures 2F–H).

Because the inhibition of HCV by lncATV knockdown could be attributed to either virus specific blocking or stimulation of host antiviral status. Therefore, we further selected several other RNA viruses, including Zika virus (ZIKV), Newcastle disease virus (NDV), and Sendai virus (SeV), for further assessment. ZIKV is a positive strand RNA flavivirus and has recently attracted global attention. As shown in Supplementary Figures 3A–C, results by testing the intracellular ZIKV RNA copies, viral envelope (E) protein levels and virus infection foci formation units showed that disruption of lncATV expression consistently impaired the ZIKV propagation in Huh7 cells. Comparatively, knockdown of lncATV suppressed NDV by decreasing viral RNA, proteins, and NDV-induced cell fusion (Supplementary Figures 3D–G). A similar regulation of lncATV to SeV was also confirmed (Supplementary Figure 3H). These findings indicate that lncATV is required for multiple virus infection probably using a universal regulatory mode.

### Regulation of lncATV on the Host Innate Immune Response

**Figure 3:**
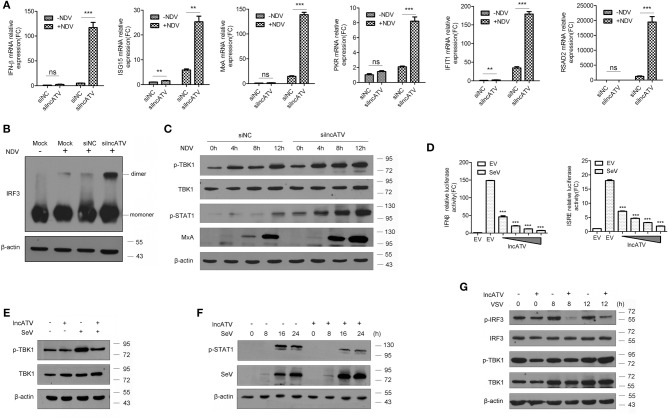
Regulation of lncATV on the host innate immune response. **(A)** Huh7 cells were transfected with negative control siRNA (siNC) or siRNA targeting lncATV at 48 h prior to infection. NDV (MOI = 0.01) was inoculated and allowed for another 16 h infection to trigger innate immune response. Total RNA was isolated, and qRT-PCR was performed to measure the expressions of IFNβ, ISG15, MxA, PKR, IFIT1, and RSAD2. **(B)** Dimerization of IRF3 was measured by native-PAGE and Western blotting. **(C)** Immunoblot analysis of phosphorylated proteins in lncATV knockdown and control Huh7 cells infected with NDV for indicated hours. **(D)** IFNβ or ISRE promoter-driven reporter construct was co-transfected with either lncATV overexpressing plasmid or empty vector (EV) into HEK293T cells. Luciferase activities were determined in the absence or presence of SeV infection. **(E,F)** HEK293T cells were transfected with lncATV overexpressing plasmid or empty vector as controls and then challenged with SeV (MOI = 1) for the indicated time courses. Phosphorylated TBK1 **(E)** or STAT1 **(F)** was detected by Western blotting. **(G)** LncATV or control empty vector was overexpressed in Raw264.7 macrophage cells. The cells were infected with VSV (MOI = 0.5) for the indicated time courses and phosphorylated IRF3 and TBK1 were detected. Typical photographs of one representative experiment were shown. Data are representative of three independent experiments and plotted as the mean ± s.d. ^**^P < 0.01, ^***^P < 0.001 vs. the control group. NS, not significant.

The broad-spectrum antiviral effects demonstrated by lncATV disruption had drawn our attention whether innate immune pathways might involve in this regulation. Therefore, we assessed the related pathways after knocking down or over-expressing lncATV in Huh7 cells. As expected, silencing lncATV by RNAi strongly enhanced the NDV-mediated induction of both IFNβ and ISGs, including ISG15, MxA, PKR, IFIT1, and RSAD2 (Figure 3A). IRF3 is a member of the interferon regulatory transcription factor family and its phosphorylation and dimerization are critical for producing IFNs. As shown in Figure 3B, NDV-stimulated IRF3 dimerization was significantly enhanced by lncATV disruption. Similar enhancements were also observed in the phosphorylation of TBK1 and STAT1 (Figure 3C). We then examined the changes of innate immune pathways in the cells over-expressing lncATV. A series of results demonstrated that lncATV antagonized IFN production and host antiviral innate immune defense. First, we used promoter-driven reporter genes, including IFNβ and ISRE, as readouts to test. These reporter genes were highly stimulated by SeV infection. However, transfection of lncATV over-expression plasmid exerted adverse impacts on reporter gene activities (Figure 3D). Western blotting data indicated that over-expressed lncATV antagonized virus-stimulated phosphorylations of TBK1, STAT1, and IRF3 (Figures 3E–G). Surprisingly, we saw a basal activation of phosphorylated TBK1 and IRF3 at 0 h without stimuli, which should not occur theoretically. Therefore, we used multiple cell lines with or without SeV infection to check in practice. Interestingly, by detecting phosphorylated TBK1, a detectable basal level of TBK1 phosphorylation could be observed in 293T, Huh7, HeLa, and HepG2 cells, but not in Hep3B cells. This phenomenon suggests that there might be some factors in cell culture conditions to maintain a low-level activation of these pathways. Our results suggest that lncATV specifically suppresses type I IFN production and its antiviral effect.

### LncATV Negatively Regulates RIG-I-Mediated Antiviral Signaling

**Figure 4:**
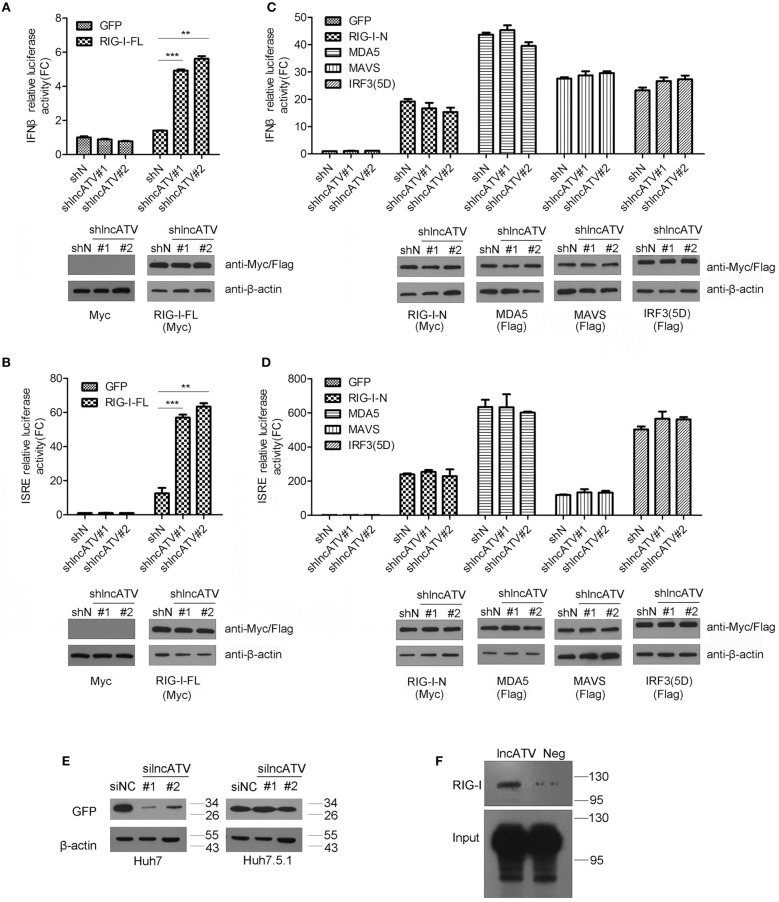
LncATV negatively regulates RIG-I-mediated antiviral signaling. **(A,B)** IFNβ **(A)** or ISRE **(B)** promoter-driven reporter activities were determined in Huh7 cells that were transfected with lncATV silencing and full-length RIG-I (RIG-I-FL) vectors. A GFP expression vector was transfected as control (upper panel). The expression of myc-tagged RIG-I was confirmed with Western blotting (lower panel). **(C,D)** The experimental design was similar to that of **(A)**, except that plasmids encoding other RLR pathway component (N-terminal RIG-I, MDA5, MAVS, or IRF3) were used instead of the overexpression of full-length RIG-I. **(E)** Huh7 or RIG-I deficient Huh7.5.1 cells were transfected with negative control siRNA (siNC) or siRNA targeting lncATV at 48 h prior to NDV-GFP infection. NDV replication levels were determined by Western blotting using anti-GFP antibody at 16 h post-infection. **(F)** RNA pull-down analysis of the binding of biotinylated lncATV to the overexpressed RIG-I in Huh7 cells. GFP sequence was transcribed and labeled as a negative control (Neg). Data are representative of three independent experiments and plotted as the mean ± s.d. ^**^P < 0.01, ^***^P < 0.001 vs. the control group. NS, not significant.

RIG-I-like receptor (RLR) pathways are major defense cascades against RNA viruses. To further define the mechanism of lncATV affecting virus replication and innate immune response, we first investigated the effect of lncATV on RLR pathways. Knockdown of lncATV acted to significantly increase IFNβ promoter activity in Huh7 cells with the transfection of full-length RIG-I but not N-terminal RIG-I, MDA5, MAVS, or the constitutively activated IRF3(5D) (Figures 4A,C). Highly consistent results were obtained when the ISRE reporter was used (Figures 4B,D). These findings indicate that lncATV may act as a potent negative regulator of RIG-I activation in the induction of type I IFNs and antiviral response. Importantly, RIG-I might be the potential target of lncATV. To further confirm this hypothesis, we compared the antiviral effects of lncATV depletion in wild type Huh7 cells and Huh7.5.1 cells that harbor a mono-allelic mutation in the CARD of RIG-I preventing its activation. Knockdown of lncATV completely lost capability to inhibit NDV replication in Huh7.5.1 cells (Figure 4E). LncRNAs function in a variety of ways, one of which is through binding to proteins (14, 17). We further determined whether lncATV was able to bind to RIG-I by RNA immunoprecipitation (RIP) technique. RIP results revealed that lncATV associated with full-length RIG-I (Figure 4F).

## Discussion

Dynamic gene expression during host antiviral response is tightly coordinated by transcriptional and post-transcriptional mechanisms. An emerging theme is the increasing important role of lncRNAs in the regulation of innate immunity (21, 22). RIG-I is a cytoplasmic sentinel that primarily detects viral RNA molecular signatures (23–25). Recent report has highlighted the importance of lnc-Lsm3b in regulating innate immune cascades in mouse macrophages by association with RIG-I (14). However, because of the species specificity of lncRNA expression, lnc-Lsm3b homologous gene cannot be found in the human genome. Even though more and more lncRNAs have been identified in various biological systems, they are mostly unexplored for the functional link with host antiviral response.

In this study, high-throughput transcriptome analysis has allowed us to identify differentially expressed human lncRNAs in response to type I and type III IFNs, which are major cellular defense effectors against virus infection. These IFN-regulated lncRNAs are valuable for future studies about infection, inflammation, and even cancer biology. With a functional screening of the top ranked differentially expressed lncRNAs, we focused on the IFN up-regulated lncATV. We provide experimental evidence showing that LncATV as a novel regulatory lncRNA to limit the innate immune response and promote viral infection. Crucially, our results show that lncATV acts through targeting RIG-I for the negative regulation of cell antiviral response.

RIG-I is considered as an important PRR to percept 5′-triphosphate dsRNA produced by pathogens for a long time (26, 27). Until recently, lncRNAs and circular RNAs were added to the list to that RIG-I bound (14, 28). However, the reason why RIG-I recognizes some “self-RNAs” remain largely unknown. Emerging evidence is revealing underlying mechanisms. Chiang et al. identified a panel of host-derived RNA binders of RIG-I after virus infection by RNA sequencing, in which the most abundant candidates were non-coding RNAs. Their findings illustrated that cellular 5S rRNA pseudogene RNA5SP141 bound to and activated RIG-I during infection with HSV-1, suggesting that innate immunity can be triggered by signals that follow pathogen assault and originate from the host itself (29). Importantly, the RNA-rich cellular environment was suggested to suppress aberrant activation of innate immunity signaling. Ahmad et al. recently reported a diverse mechanism by which endogenous RNAs act as kind of a rheostat of the innate immune system through interacting with MDA5, another RLR family member dsRNA helicase, in the context of Aicardi-Goutières syndrome (30). These findings imply that host RNAs, especial non-coding RNAs, may balance an appropriate activation or suppression status of innate immunity in virus infection and inflammatory diseases. From our microarray data, the degree of changes for lncRNAs is far less than mRNAs in response to IFN stimulation. Because tens of thousands of lncRNAs are transcribed from the human genome, they exist probably as a stable pool for the maintenance of cell homeostasis. For examples, lnc-Lsm3b inhibits RIG-I activation by competing with bona fide viral dsRNA RIG-I ligands (14). LincRNA-EPS acts as a brake to restrain uncontrolled inflammatory responses through control of nucleosome positioning and repressing transcription (31). In this study, we showed the endogenous expression of lncATV in Huh7 cells and it was further induced by virus infection and IFN treatment. However, the induced lncATV decreased host antiviral response by inhibiting the production of IFNs and ISGs. This phenomenon can be explained as a negative feedback manner to prevent excessive activation of the pathways. A similar phenotype has been reported for lnc-Lsm3b either (14). On the other hand, some lncRNAs were also reported to restrict virus infection, such as the suppressive function of the IFN-regulated lncRNA#32 in encephalomyocarditis virus, hepatitis B virus and HCV infection (32). Probably, these positive and negative regulations by lncRNA maintain a balanced antiviral immune activation status.

Taken together, we reveal a novel molecular mechanism, in which host self LncATV associates with RIG-I and inhibits RIG-I-mediated innate immunity activation. This unique regulation mode helps to understand the precise control of host immune homeostasis.

## Data Availability

All datasets generated for this study are included in the manuscript and/or the Supplementary Files.

## Author Contributions

JF and MC: generation and analysis of data and writing of the manuscript. XC and XL: validation of data. WY: study supervision, design of the experiments, and writing of the manuscript.

### Conflict of Interest Statement

The authors declare that the research was conducted in the absence of any commercial or financial relationships that could be construed as a potential conflict of interest.
