# Supplementary material for: A Human Long Non-coding RNA LncATV Promotes Virus Replication Through Restricting RIG-I–Mediated Innate Immunity
Source: Front Immunol. 2019 Jul 19;10:1711. doi: 10.3389/fimmu.2019.01711 (PMC6658999; doi:10.3389/fimmu.2019.01711)
Supplement: Supplementary file 1 [file Data_Sheet_1.docx]

**Supplementary information**

# Materials and methods

**Cells and Reagents**

Huh7, Huh7.5.1, BHK-21, Vero, Hep2, Hep3B, HepG2, Hela, Caco-2 and HEK293T were cultured in Dulbecco’s Modified Eagle’s Medium (DMEM) supplemented with penicillin and streptomycin, 1% NEAA and 10% fetal bovine serum (FBS) (Thermo Fisher Scientific, Waltham, MA, USA). RAW264.7, THP-1 and K562, were cultured in RPMI 1640 medium (Thermo Fisher Scientific) supplemented with penicillin and streptomycin and 10% FBS. HUVEC and A549 cells were maintained in F-12K medium (Thermo Fisher Scientific). All cells were incubated at 37°C in a humidified atmosphere containing 5% CO_2_. Antibodies were obtained from Cell Signaling Technology (Boston, MA, USA) for p-IRF3, p-STAT1, p-TBK1 and TBK1, Santa Cruz (Dallas, TX, USA.) for IRF3 and GFP, Millipore (Darmstadt, Germany) for RIG-I, Sigma-Aldrich (St. Louis, MO, USA) for Flag, myc and β-actin, MBL (Nagoya, Japan) for SeV, Abcam (Cambridge, UK) for STAT1, MxA and NDV, Thermo Fisher Scientific for HCV core, Biofront Technologies (Tallahassee, FL, USA) for zika virus (ZIKV) non-structural protein 1 (NS1) and envelope protein (E), respectively. Fluorescence or Horseradish peroxidase (HRP)-conjugated secondary antibodies were obtained from Jackson ImmunoResearch (West Grove, PA, USA). Peginterferon-alpha 2b (IFNα2b) was obtained from Schering Plough (Kenilworth, NJ, USA). Recombinant human IFN-lambda 1 (IFNλ1) was purchased from R&D Systems (Minneapolis, MN, USA).

**Virus infection**

The productions and infections of cell culture-derived hepatitis C virus (JFH-1) and firefly luciferase expressing HCVcc (Jc1-Luc) have been previously described (Cheng et al., 2018). Sendai virus (SeV) and Newcastle disease virus (NDV) with green fluorescent protein (NDV-GFP) was kindly provided by Dr. Zhendong Zhao (Chinese Academy of Medical Sciences, Beijing, China). SeV and NDV-GFP were propagated in 10-day-old chicken embryos, and virus was collected after infection 2 days. The titer was determined by plaque assay in LLC-MK2 cells and calculated to MOI per mL. Zika virus (ZIKV) strain SZ01 was kindly provided by Dr. Chengfeng Qin (State Key Laboratory of Pathogen and Biosecurity, Beijing, China). ZIKV stock was propagated in Vero cells, and the supernatants were collected at 72 h. The titer of Vero-amplified ZIKV was determined in target cells with serially diluted virus. Immunofluorescence focus-forming units (FFU) assay was performed at 48 h post infection and the titer was calculated to multiplicity of infection (MOI) per mL. Recombinant VSV with GFP (VSV-GFP) was kindly provided by Dr. Hui Xiao (Institut Pasteur of Shanghai, Chinese Academy of Sciences, Shanghai, China). VSV-GFP was propagated in BHK-21 cells. The titers of virus stocking supernatant were determined by standard foci formation unit assays (FFU) for HCV, SeV, NDV-GFP and ZIKV, or plaque formation unit (PFU) assay for VSV-GFP. For virus infections, the inoculation dosage and infection duration were optimized according to experimental purposes. To determine virus infection, viral RNAs were measured with qRT-PCR and viral protein or reporter protein were detected by Western blotting and immunofluorescent foci formation unit assays.

**Plasmids**

LncATV was amplified from Huh7 cells using the SuperScript III First-Strand Synthesis System (Invitrogen, Carlsbad, CA, USA). LncATV cDNA was cloned into pSilencer-CMV-4.1 after BamHI and HindIII digestion. The cloning primers were listed in Supplementary Table 1. The shRNA targeting lncRNAs were cloned into pQsuper-R plasmid vector after BglII and XhoI digestion. The shRNA targeting sequences were same as siRNAs sequences, as listed in Supplementary Table 1. The plasmids RIG-I (Myc), MDA5 (Flag), MAVS (Flag), IRF3 (5D) and the IFNβ and ISRE luciferase reporter plasmids were kindly provided by Dr. Zhendong Zhao.

**RNA interference**

All the small interfering RNAs (siRNA), including the scrambled negative control siRNA (siNC), were purchased from RiboBio (Guangzhou, China). The siRNAs sequences for lncATV were listed in Supplementary Table 1. Transfection of siRNA was performed using Lipofectamine RNAiMAX (Invitrogen) following the manufacturer’s instruction.

**RNA pull-down assay**

LncATV RNA and GFP-coding RNA as a negative control were *in vitro* transcribed and biotin-labeled with the Biotin RNA Labeling Mix (Roche Diagnostics, Indianapolis, IN), and purified with an RNeasy Mini Kit (Qiagen, Valencia, CA). The biotinylated lncATV probe was incubated with Dynabeads M-280 Streptavidin (Invitrogen) at room temperature for 10 min to generate probe-coated beads according to the manufacturer’s protocol. Empty beads were used as a negative control. RIG-I overexpression plasmid transfected cell lysates were incubated with the probe-coated beads, and the RNA complexes bound to these beads were eluted for Western blotting analysis.

**In-Cell Western (ICW)**

ICW is created by LI-COR, a quantitative immunofluorescence assay performed in microplates (96- or 384-well format) that combines the specificity of Western blotting with the reproducibility and throughput of ELISA. It’s a kind of alternative of quantitative immunofluorescence staining or FFU assay, using spectrally-distinct infrared scanner instead of fluorescence microscope. Briefly, Huh7 cells were seeded in 96-well plate format at 1 × 10^4^ per well one day before transfection of various siRNAs. Twenty-four hours post transfection, the cells were infected with either ZIKV or NDV-GFP infection (MOI = 0.01) for another 2 days. The cells were fixed in the original wells with paraformaldehyde and permeabilized with Triton X-100, followed with immunostaining with anti-ZIKV NS1 or anti-GFP antibodies (1:400 dilution) and IRDye Secondary Antibody (1:1000 dilution, Li-Cor, Nebraska, USA). Images were obtained on Odyssey Infrared Imaging System (Li-Cor, Lincoln, NE, USA).

**Luciferase reporter assay**

Huh7 or HEK293T cells were seeded in 24-well plates, and the transfection was processed using PEI (Sigma) when the cell confluent at 70-90%. The total amount of DNA was kept constant by adding empty control plasmid. The cells were lysed to determine luciferase activities using luciferase reporter assay system (Promega, Madison, WI, USA).

**Quantitative real-time reverse transcription-PCR (qRT-PCR)**

Total cellular RNA was isolated using Trizol reagent according to the manufacturer’s protocol. One step qRT-PCR was performed using the QuantiFast SYBR Green RT-PCR Kit (Qiagen, Düsseldorf, Germany) on ABI Prism 7900 System. The primer pairs used for qRT-PCR were listed in Table 1. The relative quantification of gene expression was performed. House-keeping gene GAPDH was used as the internal normalization control. The 2^−ΔΔCT^ method was chosen for data analysis to represens the fold change of the treated target gene relative to the control. For absolute quantification in real-time RT-PCR, lncATV RNA was *in vitro* transcribed (Ambion, AM1333), purified (Ambion, AM1908), calculated to copy number and serially diluted as standards.

**Western blotting**

These experiments were performed as described elsewhere (Cheng et al., 2018). Dimerization of IRF3 was detected by native PAGE as described previously (Gack et al., 2008; Li et al., 2017). Briefly, the monolayer cells were lysed with lysis buffer (50 mM Tris-HCl, pH 7.5, 150 mM NaCl, 1% Nonidet P40, 50 mM NaF, 1 mM Na_3_VO_4_, 5 mM β-glycerophosphate, 1 mM dithiothreitol, and 1 mM phenylmethylsulfonyl fluoride) supplemented with a protease inhibitor cocktail (Sigma-Aldrich) on ice. The lysate was cleared by centrifugation at 14,000 × g for 20 min. The samples were boiled in 2× SDS loading buffer and loaded onto a 10-12% polyacrylamide gel. After electrophoresis, the separated proteins were transferred onto a nitrocellulose membrane (Bio-Rad, Hercules, CA). The resulting blots were blocked with 10% milk for 1 h and then incubated with a primary monoclonal antibody overnight at 4°C. After 3 washes of the blots, an HRP-linked secondary antibody was applied at a 1:1000-1:5000 dilution. The ECL reagent (Amersham Biosciences, Piscataway, NJ, USA) was used for detection.

**Confocal microscopy**

Cells cultured on glass slides were fixed with 4% paraformaldehyde in PBS at room temperature for 30 min. After two washes with PBS, fixed cells were permeabilized with 0.1% TritonX-100 in PBS for 5 min and were incubated with an HCV anti-core antibody overnight at 4°C (Si et al., 2012). After three washes with PBS, cells were further incubated with DyLight fluorescent dyes (Thermo Fisher Scientific, Inc., USA) for 1hr at room temperature. Nuclei were stained with 4’,6-diamidino-2-phenylindole (DAPI). After two washes with 0.1% Triton X-100 in PBS and three washes with PBS, cells were analyzed using the Zeiss LSM 700 laser confocal microscopy system (Carl Zeiss, Inc., Thornwood, NY).

**Fluorescence in situ hybridization**

Huh7 cells cultured on glass slides were fixed in 4% paraformaldehyde and then permeabilized with 0.5% TritonX-100. Then cells were hybridized with lncATV probe using the ViewRNA™ ISH Cell Assay Kit (Thermo Fisher Scientific) according to the manufacturer’s protocol. The detailed protocol was described elsewhere (Wang et al., 2017). The samples were visualized using Leica TCS-SL confocal microscope.

**Statistical Analysis**

The values shown in graphs are presented as the mean ± standard deviation. Data are representative of at least three independent experiments with similar results. Data were analyzed using GraphPad Prism 6.01 (GraphPad Software, San Diego, California, USA). Statistical differences between groups were analyzed using a one-way ANOVA statistical test with Dunnett multiple comparisons tests; p < 0.05 was considered statistically significant.

**Supplementary table 1. List of primers and siRNA sequences.**

| **Primer/siRNA name** | **Sequences** | **Purpose** |
| --- | --- | --- |
| lncATV-Forward | 5’-CGGGATCCTCTATTCTTGTAGATTTTTTTTGTGTGG-3’ | lncATV cloning |
| lncATV-Reverse | 5’-CCAAGCTTTAGAGAGATAAAAGTTTTATTTCATAAGTA-3’ | lncATV cloning |
| silncATV#1 | 5’-GGAGTTGAAAGCGCAGCTT-3’ | lncATV knockdown |
| silncATV#2 | 5’-GAGCAAGATTCCAACATCA-3’ | lncATV knockdown |
| qGAPDH-Foward | 5’-TGTCCCCACCCCCAATGTATC-3’ | qRT-PCR |
| qGAPDH-Reverse | 5’-CTCCGATGCCTGCTTCACTACCTT-3’ | qRT-PCR |
| qMxA-Foward | 5’-TGGAGATCAGCTCCCGAGATG-3’ | qRT-PCR |
| qMxA-Reverse | 5’-ATTGCCCACAGCCACTCTG-3’ | qRT-PCR |
| qIFIT1-Foward | 5’-GCCTTGCTGAAGTGTGGAGGAA-3’ | qRT-PCR |
| qIFIT1-Reverse | 5’-ATCCAGGCGATAGGCAGAGATC-3’ | qRT-PCR |
| qISG15-Foward | 5’-CTCTGAGCATCCTGGTGAGGAA-3’ | qRT-PCR |
| qISG15-Reverse | 5’-AAGGTCAGCCAGAACAGGTCGT-3’ | qRT-PCR |
| qPKR-Foward | 5’-GAAGTGGACCTCTACGCTTTGG-3’ | qRT-PCR |
| qPKR-Reverse | 5’-TGATGCCATCCCGTAGGTCTGT-3’ | qRT-PCR |
| qRSAD2-Foward | 5’-TGCTTTTGCTTAAGGAAGCTG-3’ | qRT-PCR |
| qRSAD2-Reverse | 5’-AGGTATTCTCCCCGGTCTTG-3’ | qRT-PCR |
| qIFNβ-Foward | 5’-GATTCATCTAGCACTGGCTGG-3’ | qRT-PCR |
| qIFNβ-Reverse | 5’-CTTCAGGTAATGCAGAATCC-3’ | qRT-PCR |
| qlncATV-Foward | 5’-AAACCTAACCCTCGCACGCCAA-3’ | qRT-PCR |
| qlncATV-Reverse | 5’-AGCTATGTCGCACAGGAATGGCATC-3’ | qRT-PCR |
| qHCV 5’UTR-Foward | 5’-GTCTAGCCATGGCGTTAGTA-3’ | qRT-PCR |
| qHCV 5’UTR-Reverse | 5’-CTCCCGGGGCACTCGCAAGC-3’ | qRT-PCR |
| qNDV-N-Foward | 5’-ACTCAAGAGAGGCCGCAATAC-3’ | qRT-PCR |
| qNDV-N-Reverse | 5’-AGTGCAAGGGCTGATGTCTT-3’ | qRT-PCR |
| qZIKV-Foward | 5’-GGTCAGCGTCCTCTCTAATAAAC-3’ | qRT-PCR |
| qZIKV-Reverse | 5’-GCACCCTAGTGTCCACTTTTTCC-3’ | qRT-PCR |

**Supplementary table 2. Homology comparison of lncATV from different species.**

| species | Coverage | similarity |
| --- | --- | --- |
| Human | 100% | 100% |
| Pan troglodytes | 63% | 99% |
| Gorilla gorilla gorilla | 51% | 99% |
| Papio anubis | 99% | 95% |
| Macaca fascicularis | 99% | 95% |
| Cebus capucinus | 41% | 85% |
| Microcebus murinus | 58% | 72% |
| Callithrix jacchus | 17% | 89% |
| Bos mutus | 79% | 69% |
| Ovis canadensis canadensis | 74% | 67% |
| Nomascus leucogenys | 9% | 96% |
| Sus scrofa | 64% | 66% |
| Mustela putorius furo | 16% | 70% |
| Mus musculus | 6% | 81% |
| Mouse | 3% | 81% |
| Zebrafish | 3% | 83% |

**Supplementary figures**


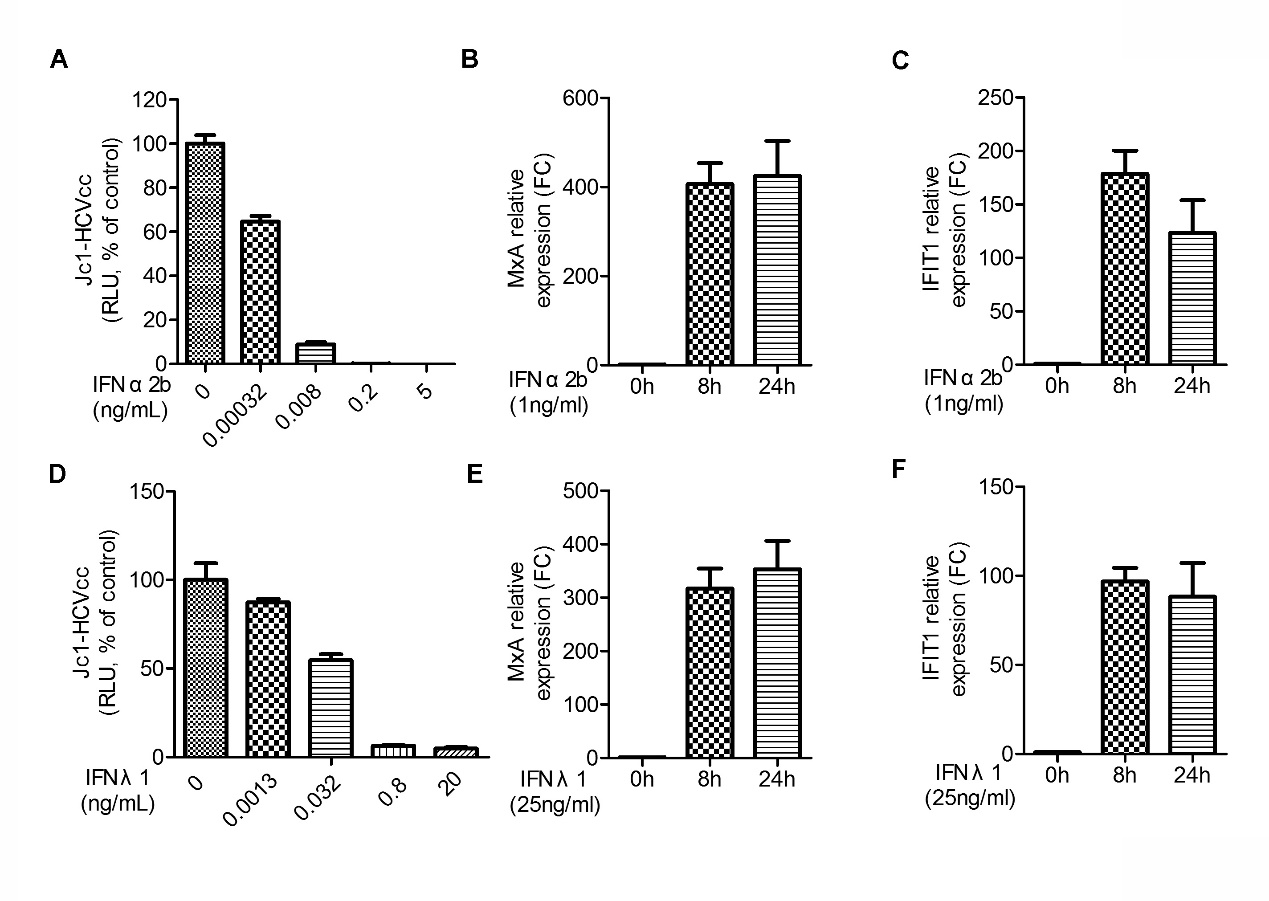


**Supplementary Figure 1. Optimization of IFN treatment concentrations.** (**A, D**) Huh7 human hepatoma cells were infected with Jc-1 reporter HCVcc (MOI=0.5) for 24 h and then treated with either IFNα2b (A) or IFNλ1 (D) at various concentrations for another 48 h. The cells were lysed, and luciferase activity was assayed. (**B, E**) Huh7 cells were treated with 1 ng/mL IFNα2b (B) or 25 ng/mL IFNλ1(E) for different time courses. Cellular total RNAs were isolated for quantification of MxA mRNA expression and described as relative fold change (FC). (**C, F**) The relative expression of IFIT1 mRNA was determined by qRT-PCR in response to IFNα2b (C) or IFNλ1(F) using the similar experimental designs as described above. Means and standard deviation (SD) of results are from three independent experiments in triplicate. RLU is relative light units.


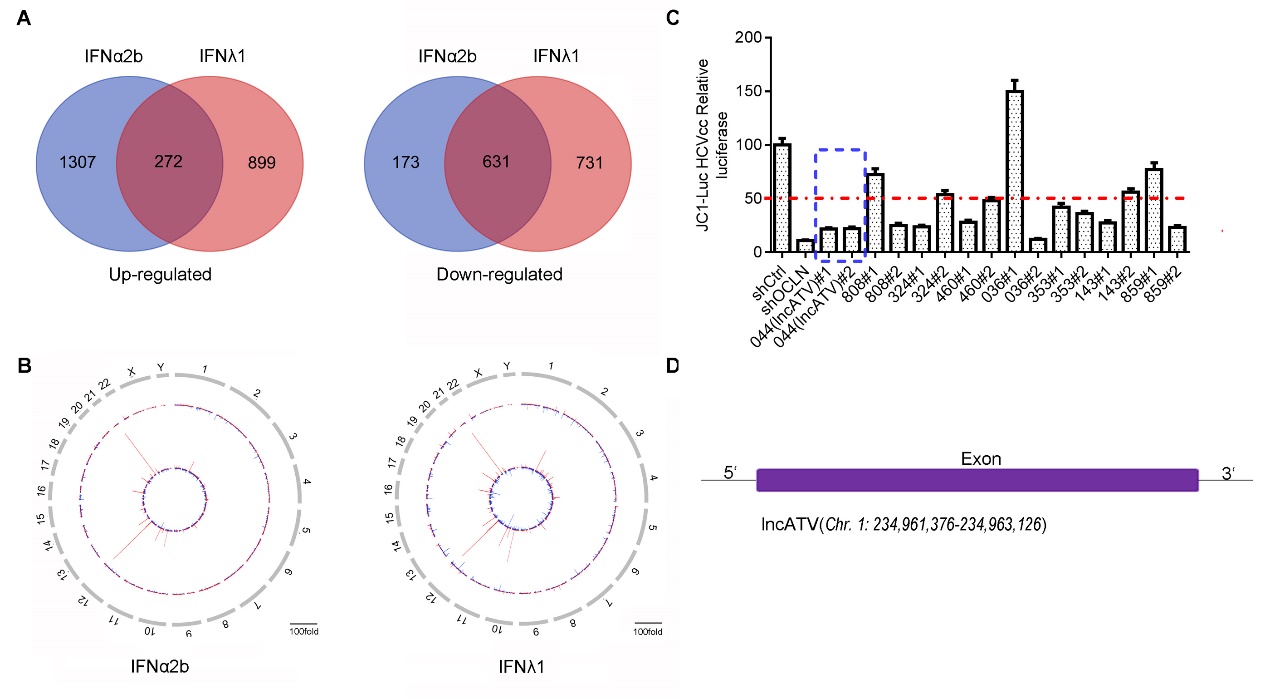


**Supplementary Figure 2. Identification of IFN-regulated lncRNAs.** (**A**) Venn diagram of differentially expressed lncRNAs (>2.0-fold) by IFNα2b and IFNλ1. (**B**) Representative circos plot demonstrates differentially expressed lncRNAs (outer track) and mRNAs (inner track) in chromosomes. Red indicates up-regulated genes; blue is down-regulated genes. (**C**) Top-ranked differentially expressed lncRNAs were selected for identification of their potential regulation on HCV infection. LncRNA-specific siRNAs were transiently transfected into Huh7 cells. HCVcc carrying firefly luciferase reporter gene was inoculated at 24 h post siRNA transfection and allowed for another 48 h infection. Relative luciferase units were determined. SiRNAs targeting HCV entry co-receptor Occludin (OCLN) were used as positive controls. (**D**) Genome organization of lncATV.


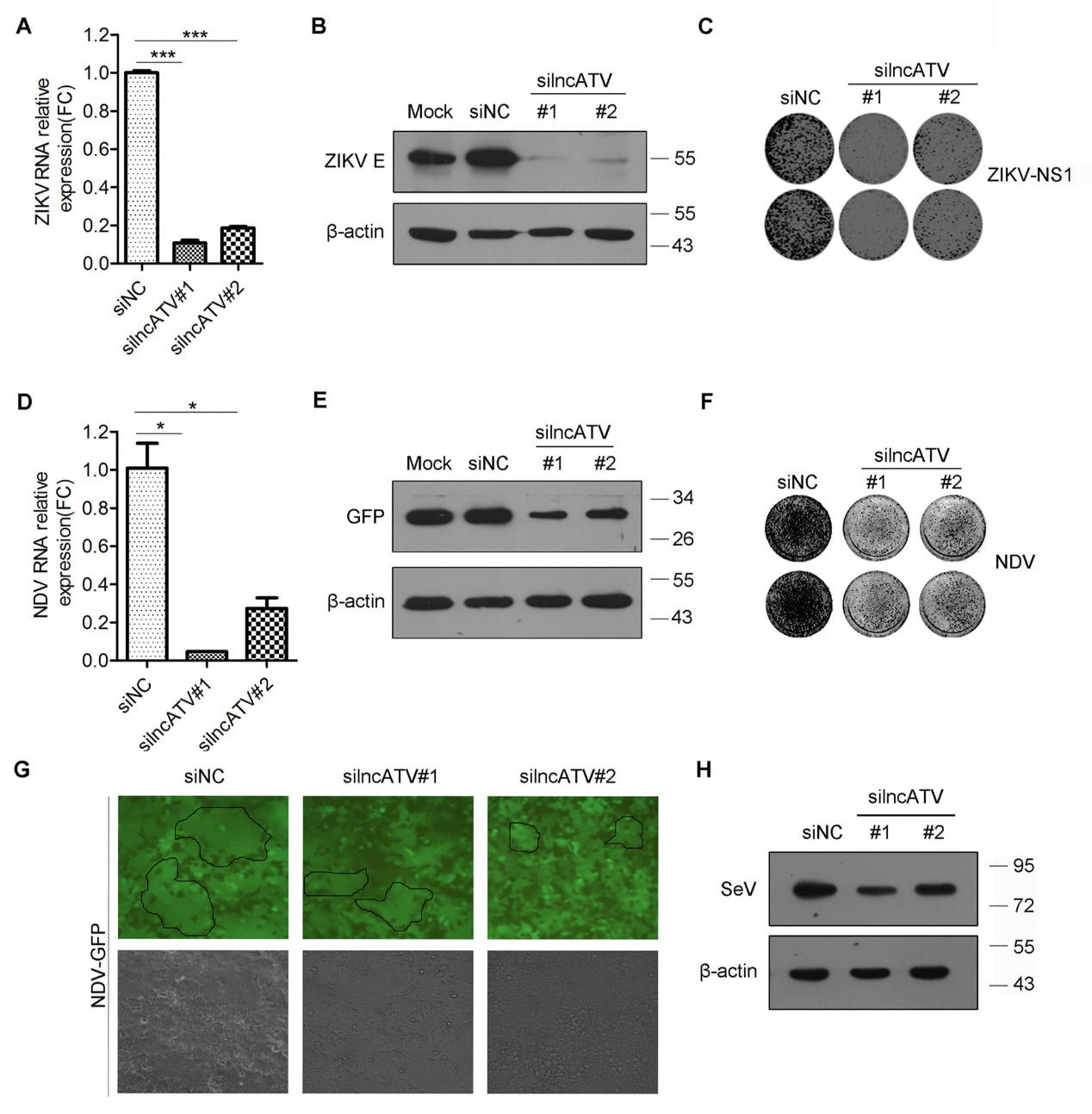


**Supplementary Figure 3. LncATV is required for the infection of multiple RNA viruses.** (**A, B, C**) Silencing lncATV significantly inhibits ZIKV infection. Huh7 cells were transfected with negative control siRNA (siNC) or siRNAs targeting lncATV at 48 h prior to ZIKV infection. The infection was allowed for another 48 h. The efficiency of ZIKV infection was determined by qRT-PCR for viral RNA (A), Western blotting for viral E protein (B) and immunostaining for virus foci formation (C). (**D, E, F, G**) Knockdown of lncATV suppresses NDV infection. Intracellular NDV viral RNA was detected by qRT-PCR (D), NDV encoded GFP protein was measured by Western blotting (E), NDV infection foci formation was determined by immunostaining (F), and NDV HN protein induced cell fusion (circled with black line) was also visualized (G). (**H**) Silencing lncATV inhibits SeV infection in Huh7 cells. Data are representative of three independent experiments and plotted as the mean ± s.d. *, P <0.05, ***, P <0.001 vs. the control group.
